# Editorial: Novel brain imaging methods for the aid of neurological and neuropsychiatric disorders

**DOI:** 10.3389/fnins.2024.1468794

**Published:** 2024-08-07

**Authors:** Takao Yamasaki, Zhiyong Zhao

**Affiliations:** ^1^Department of Neurology, Minkodo Minohara Hospital, Fukuoka, Japan; ^2^Kumagai Institute of Health Policy, Fukuoka, Japan; ^3^Children's Hospital, Zhejiang University School of Medicine, National Clinical Research Center for Child Health, Hangzhou, China

**Keywords:** brain imaging methods, neuroimaging, neurological and neuropsychiatric disorders, biomarkers, analytical methods, therapeutic application

## 1 Introduction

The human brain is a highly complex and dynamic system comprising extensive structural and functional networks connecting different brain regions, operating at multiple spatial and temporal scales (Bassett and Bullmore, 2009; Bullmore and Sporns, 2009). This network system is the basis for our daily activities and cognitive functions, and its disruptions can cause various neurological and neuropsychiatric disorders, including Alzheimer's disease, Parkinson's disease, major depressive disorder (MDD), schizophrenia (SZ), and autism spectrum disorder (ASD) (Yamasaki et al., 2017; Miraglia et al., 2022; Cattarinussi et al., 2023; Tura and Goya-Maldonado, 2023).

Neuroimaging techniques, such as electroencephalography (EEG), magnetoencephalography (MEG), functional near-infrared spectroscopy (fNIRS), and magnetic resonance imaging (MRI), play a vital role in examining the healthy and pathological functions of the human brain network system. These techniques allow us to explore the brain at different temporal and spatial scales, taking advantage of the unique characteristics of each method (Yen et al., 2023; Zhu et al., 2023). Moreover, with the rapid development and widespread use of these neuroimaging techniques, the image analysis method has made considerable advances (Zhang et al., 2020) to help us in clarifying the pathological mechanism, early diagnosis, and complementary treatment of various clinical disorders.

Therefore, the purpose of this Research Topic is to collect data on the latest biomarkers, analytical methods, and therapeutic applications that will be useful for treating neurological and neuropsychiatric disorders.

## 2 Research on neuroimaging biomarkers

### 2.1 MRI

Among the various types of MRI techniques, diffusion tensor imaging (DTI) and resting-state functional MRI (rs-fMRI) have been widely used to investigate the structural and functional connectivity of the brain, respectively (Zhu et al., 2023). A significant advantage of MRI techniques is their excellent spatial resolution (Bassett and Bullmore, 2009).

This Research Topic includes one study on DTI and three studies on rs-fMRI biomarkers. In the DTI study, Zheng et al. revealed that DTI analysis along the perivascular space index, possibly indicating glymphatic activity, might be useful as a new biomarker for the early diagnosis of radiation encephalopathy. Regarding the rs-fMRI studies, Cheng et al. observed that the functional connectivity of the corticobasal ganglia network was altered in patients with idiopathic blepharospasm and correlated with disease severity, thus indicating its potential use as a quantitative marker of disease severity. Li Y. et al. showed frequency-specific alterations in causal influences among triple networks (i.e., default mode network, salience network, and central executive network) in patients with MDD, which might be useful as accurate and reliable biomarkers for MDD. Furthermore, Zhu et al. demonstrated that first-episode and recurrent MDD exerted distinct effects on the effective connectivity among large-scale brain networks, which might be potential neural mechanisms underlying the different clinical manifestations for the two MDD subtypes.

### 2.2 fNIRS

fNIRS is an optical neuroimaging technique used to image hemodynamic activity and connectivity in the brain and has better temporal resolution than fMRI (Pinti et al., 2020).

This Research Topic includes two studies on fNIRS biomarkers. Peng et al. showed that compared with that in the resting-state, brain network properties in the task-state were significantly different between poststroke depression (PSD) and non-PSD groups, resulting in improved classification performance. These findings demonstrated the feasibility and superiority of brain network properties in the task-state for exploring the neural mechanisms of PSD. Wu et al. found that patients with short-term insomnia disorder (SID) exhibited an aberrant functional connectivity pattern in the prefrontal cortex during the verbal fluency test task, which correlated with the severity of sleep disturbances. Hence, fNIRS can contribute to the early detection and diagnosis of patients with SID, thereby effectively reducing the risk of disease progression.

### 2.3 EEG and MEG

EEG and MEG signals are more directly related to neuronal activity and have more excellent temporal resolution than fMRI and fNIRS (Bassett and Bullmore, 2009; Gross, 2019).

This Research Topic includes one study each on EEG and MEG. Using a 128ch EEG system, Liu et al. recorded auditory evoked potential in patients with chronic fatigue syndrome (CFS) and demonstrated the significant correlation between the P50 sensory gate ratio and clinical symptoms such as fatigue, anxiety, and depression. The P50 sensory gate ratio may be remarkably used for clarifying the mechanism, classification, treatment, and prognosis of CFS. In the MEG study, Nakanishi et al. reported that the abnormal phase lead on 80 Hz auditory steady-state response exhibited the highest discriminative power between patients with SZ and healthy individuals. They concluded that this testing technique has significant potential as a strong candidate for identifying neurophysiological endophenotypes associated with SZ.

## 3 Research on neuroimaging analysis methods

ROI-based and data-driven methods are two common approaches used to analyze functional connectivity derived from rs-fMRI data. The ROI-based method requires prior knowledge of targeted regions and consists of statistical parametric mapping, coherence analysis, and cross-correlation analysis. However, the data-driven method relies on acquired data, including decomposition [clustering analysis and principal component analysis/independent component analysis (ICA)], graph theory, and machine learning (Chauhan and Choi, 2022).

This Research Topic includes two studies on state-of-the-art rs-fMRI analysis methods. Jing et al. found that both group information-guided ICA (GIG-ICA) and independent vector analysis-Gaussian-Laplacian density models (IVA-GL) demonstrated distinct capabilities in identifying brain network modules in patients with ASD and healthy subjects. GIG-ICA can detect more regions with higher amplitudes in spatial network differences, and IVA-GL can identify more networks associated with ASD. This study provides further insights into using different data-driven methods to investigate neurological disorders using rs-fMRI. In the other study, Li W.-X. et al. showed that directed functional connectivity quantified using a new complex-valued transfer entropy (CTE) method had higher classification accuracy between patients with SZ and healthy subjects than other methods. Therefore, their proposed CTE provides a new general method for fully detecting highly predictive directed connectivity from complex-valued fMRI data.

## 4 Research on the application of neuroimaging to rehabilitation and treatment

This Research Topic includes two studies on the application of neuroimaging to rehabilitation and treatment. Neurofeedback using neuroimaging techniques (e.g., EEG, MEG, and fMRI) has been used as a cognitive training tool to improve brain functions (Loriette et al., 2021). Takahashi et al. developed a portable, wearable NIRS-based neurofeedback system and demonstrated the usefulness of this system for older adults and its potential to reduce cognitive decline. Electrical stimulation, such as transcranial direct current stimulation (tDCS), is widely used to treat neurological and neuropsychiatric disorders (Stagg and Nitsche, 2011). Computational modeling is an important approach for understanding the mechanisms underlying tDCS and optimizing treatment plans. Katoch et al. conducted *in vivo* magnetic resonance conductivity tensor imaging (CTI) experiments on the entire brain to precisely estimate tissue responses to electrical stimulation. They suggested that this CTI-based, subject-specific model can provide detailed information on tissue responses for personalized tDCS treatment plans.

## 5 Conclusion

This Research Topic provides information on novel brain imaging methods that can aid in the treatment of neurological and neuropsychiatric disorders, with a particular focus on the latest biomarkers, analytical methods, and therapeutic applications. We believe that this Research Topic will provide valuable insights to guide future research efforts and clinical practice.

## Author contributions

TY: Writing – original draft, Writing – review & editing. ZZ: Writing – review & editing.
